# From genes to germ layers: virtual twins of gastruloids

**DOI:** 10.1038/s41540-026-00709-z

**Published:** 2026-04-28

**Authors:** Leona M. Irwin, Shaun M. Cowley, Himanshu Kaul

**Affiliations:** 1 Division of Molecular and Cell Biology, School of Biological and Biomedical Sciences, University of Leicester, Leicester, UK; 2 Division of Respiratory Sciences, School of Medical Sciences, University of Leicester, Leicester, UK; 3 School of Engineering, University of Leicester, Leicester, UK

**Keywords:** Computational biology and bioinformatics, Developmental biology, Stem cells

## Abstract

Human gastrulation remains a black box due to ethical and technical reasons. Human pluripotent stem cell-based 2D micropatterns and 3D organoids (gastruloids) recapitulate aspects of human gastrulation. Understanding the integrated multiscale biology of gastrulation, however, remains elusive. We explain the barriers to recapitulating the multiscale biology of gastrulation in silico, how agent-driven multi-paradigm approach can overcome these challenges, provide relevant examples, and share best practices on developing the virtual twins.

## Introduction

“It is not birth, marriage, or death, but *gastrulation*, which is truly the most important part of your life”—goes the dictum by Lewis Wolpert^1^. Gastrulation represents one of the earliest milestones in human embryogenesis (and development) and yields the three germ layers (ectoderm, mesoderm, and endoderm) along with the basic body axes that together are responsible for the formation of all human tissues and organs. The phrase gastrulation originates from the Greek word “*gaster*” that translates to “belly” or “gut”. The embryonic gastrula was first described by the German embryologist Ernst Haeckel in the 1870s^2^ based on his observations of the early embryonic infolding and invagination, which subsequently formed the organism’s digestive tract with its musculature present as a separate layer inside the embryo. We now understand gastrulation as the orchestrated transformation of a seemingly homogenous and symmetrical bundle of “blastula” cells into a complicated, diverse and multi-layered embryo with an internal compass.

**Fig. 1:**
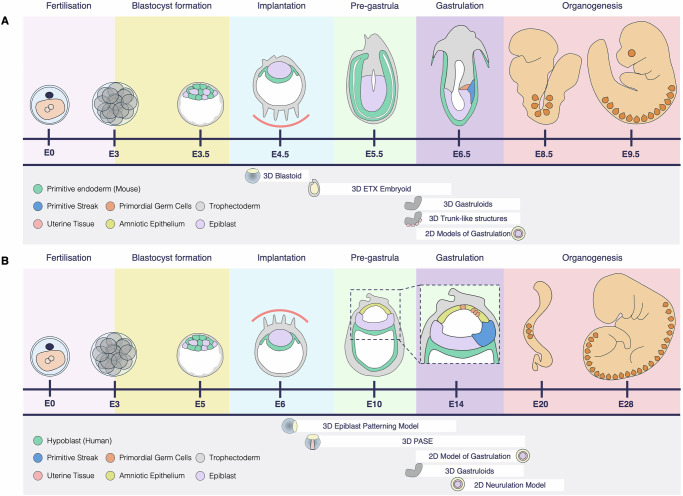
A comparison of early in vivo embryogenesis in mouse and human contexts alongside their in vitro stem cell model counterparts. **A** Mouse embryogenesis is shown from the fertilisation to the completion of organogenesis **B** Human embryonic development is shown from the fertilisation to organogenesis. ETX; Embryonic–Trophoblast–Extra-embryonic endoderm model. PASE Post-implantation Amniotic Sac Embryoid. Timelines in this figure for human, mouse, and in vitro models were integrated from elsewhere^113–115^.

During gastrulation, gene expression is under both temporal and spatial control^3^. In both human and murine development, a single cell undergoes multiple mitotic divisions to form the morula at around embryonic day (E) 3 post-fertilisation. Just after implantation (Fig. 1), in both mice and humans, gastrulation is triggered by the emergence of a population of cells identified as the primitive streak^4^. During gastrulation, the epiblast cells undergo epithelial-to-mesenchymal transition (EMT) and ingress into the primitive streak, producing both mesoderm (progenitors of muscle, cartilage and connective tissue) and endoderm (gastrointestinal tract, pancreas, etc.)^5^. Positioning of the primitive streak from posterior to anterior side of the embryo determines its eventual body axis. From the posterior side, the lateral plate mesoderm, intermediate mesoderm, anterior paraxial mesoderm, and axial mesoderm stretch out to definitive mesoderm at the anteriorly located primitive streak^6^. The epiblast cells that did not progress through the primitive streak form the ectoderm, which eventually gives rise to the nervous system and epidermis^4^.

**Fig. 2:**
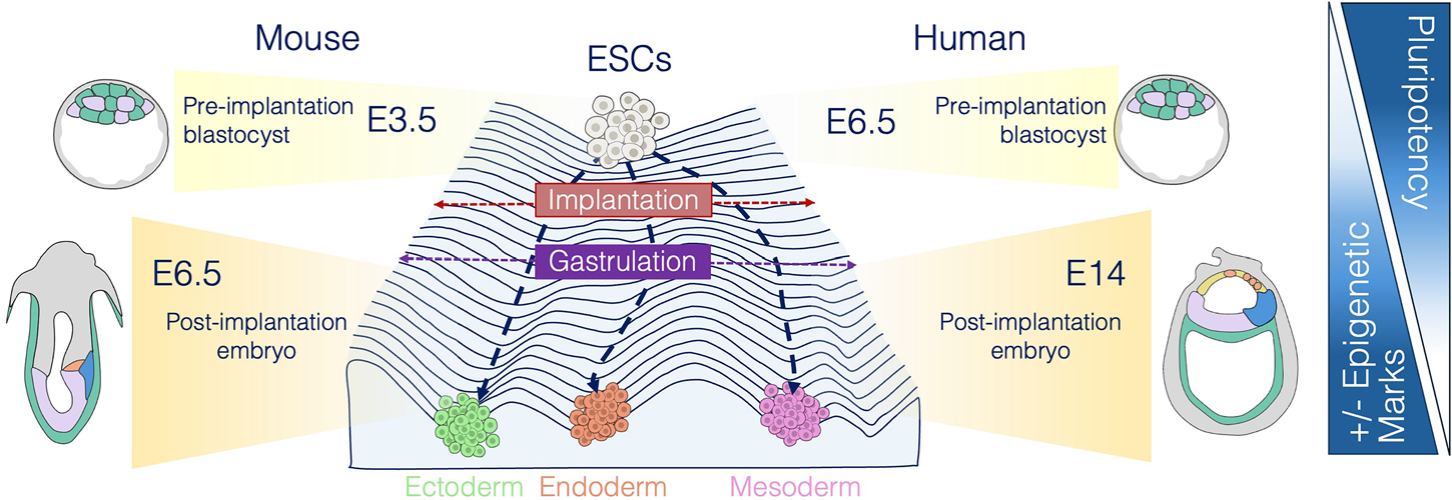
A schematic showing that the underlying epigenetic landscape can mediate cell fate during gastrulation in the style of the Waddington landscape^116^. Both mESCs (left) and hPSCs (right) from the inner cell mass lose pluripotency during gastrulation and early development generally. Both loss and gain of epigenetic markers contribute to the determination of cell fate and differentiation (ectoderm/endoderm/mesoderm). This is both decided and committed to post-implantation. Figure created using NIAID NIH BIOART^117^.

It is understood that these events are triggered and mediated by a sequence of Bone Morphogenetic Protein-4 (BMP4), Wingless-INTs-3 (WNT3), and NODAL signalling^7^. Reports suggest that this hierarchy of signalling pathways is conserved in humans^8^. In addition to signalling, efforts have been made to investigate how epigenetic changes, i.e. changes to the characteristics of a cell via alterations to the gene expression that do not involve changes to the underlying deoxyribonucleic acid (DNA) sequence^9^, regulate these patterns (Fig. 2). Alterations to the epigenetic landscape such as methylation and acetylation of histone tails are essential to the commitment of cell identity in gastrulation^10^. In fact, Foster et al.^11^ showed that the loss of lysine demethylase 1 (LSD1) at the time of gastrulation (E6.5) in mouse embryos is lethal.

Despite its biological significance, human gastrulation remains the ultimate biological black box due to legal, ethical, and technical limitations. Typically, ex vivo/in vitro investigations using human embryos are limited to 14 days post-fertilisation because in the first two weeks biological individuation has not occurred, and so an embryo can split in two or fuse together^12,13^. This so-called *14-day rule* was initially approved by the Warnock Committee in the UK^14^ in 1984 and subsequently incorporated into the 1990 UK Human Fertilisation and Embryology Act^15^. However, parallel to ethical and legal restrictions, technical challenges abound. For a start, gastrulating embryos are translucent^16^ which makes their in utero imaging near impossible. Furthermore, accurately predicting the window of implantation and embryonic gastrulation in expectant mothers, who themselves are most likely unaware they are pregnant at this moment in time, is extremely challenging.

Another critical technical barrier is the complexity of the phenomenon itself. Gastrulation emerges due to bi-directional cross-talks that exist between the cells, and cells and their microenvironment^6,17–21^. Specifically, gene regulatory networks (GRNs) influence cell interactions, which shape the local microenvironment, in turn, shaping the very same GRNs to mediate physiology and mechanics at the tissue-level^22^. These interactions are highly decentralised with no single locus origin of development. To add to this complexity, these decentralised interactions are non-linear, occur across multiple scales of space and time, and are underpinned by numerous heterogeneous components that display flexible memberships (differentiation, re-differentiation). This leads to self-organisation and adaptive behaviour, which makes understanding gastrulation challenging.

Our (basic) understanding of human gastrulation has arisen due to, and is intertwined with, the scientific innovations responsible for assisted reproductive technologies^23^. Understanding the specific mechanisms that regulate gastrulation has significant societal implications. For example, this can shed insights into the currently unknown reasons that underpin early miscarriages in both clinically complicated and atypical pregnancies. This understanding is urgently needed, given that an estimated 23 million miscarriages occur each year globally, equivalent to 44 pregnancy losses every single minute^24^. Further, of the (30%) human conceptions that progress into a successful pregnancy, 30% fail during pre-implantation and another 30% fail between weeks 2 and 6, which coincides with the gastrulation window^6,24^. Additionally, a comprehensive understanding of human gastrulation can also increase the success rate of in vitro fertilisation, currently at 30%^25^, which is the most effective^26^ recourse available to 1/6 people who experience infertility. In the UK, these issues come at a significant economic cost of £471 million per year^24^ as well as the immeasurable emotional toll on those experiencing it. Moreover, this multiscale knowledge has implications to furthering our understanding of human congenital malformations, such as conjoined twins^27^, abnormal caudal mesoderm development^28,29^, and spinal cord pathologies^30^, typically associated with irregular gastrulation. Congenital foetal abnormalities are risk factors for pregnancy complications and maternal morbidity^31^. Developing, therefore, a systems-level understanding of human gastrulation will also help reduce maternal mortality, which represents one of the Sustainable Development Goals (SDG) established by the United Nations (UN).

Computational methods, especially virtual twins^32^, represent an exciting tool to overcome this challenge. But while we have mathematical formulations to recapitulate biological decision-making at each hierarchical level (Boolean models to capture GRN networks, agent-based models for cellular interactions, continuum models for tissue/organ physiology and mechanics, statistical models at the organism level), lack of formulations that can effectively integrate the diversity of these processes (genomic, cellular, microenvironmental) remains an outstanding impediment^33^.

Here, we posit that an agent-driven^22^ multi-paradigm^34^ approach is optimal for creating virtual twins^17^ of the human embryo/stem cell-based embryo models to recapitulate the multi-modal and multiscale mechanisms that often act orthogonally, to mediate the emergence of germ layers during gastrulation. We briefly review the key in vitro and in silico models in this domain, propose agent-based multi-paradigm approach as a solution, and finally suggest “best practices” to developing these models.

### In vitro models displaying gastrulation-like patterns

**Fig. 3:**
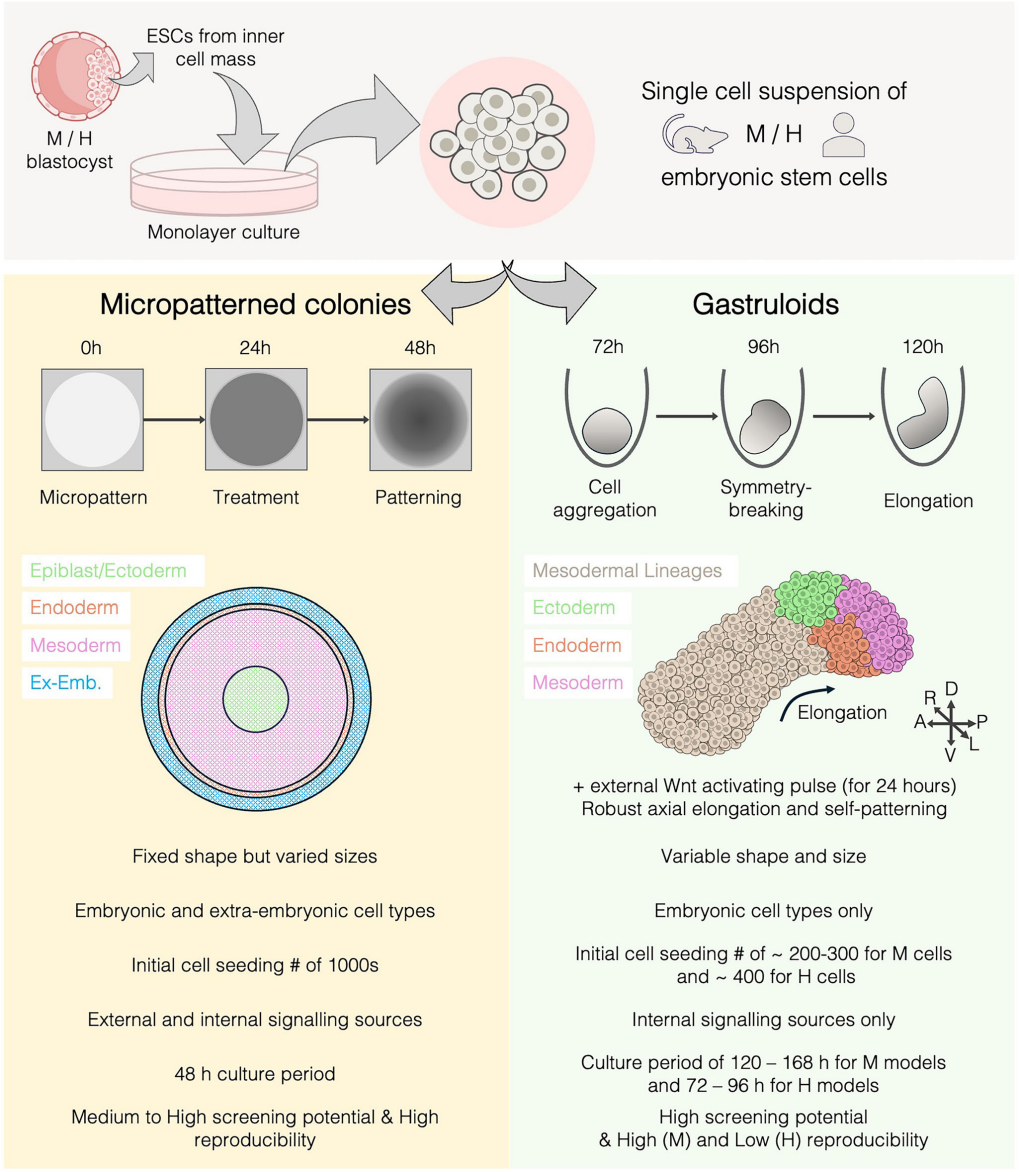
A schematic comparison of in vitro 2D and 3D stem cell-based models of early embryonic development. Both mouse and human embryonic stem cells can be cultured as 2D micropatterns or 3D organoids to recapitulate signatures of early embryonic development, including germ layer formation and acquisition of body axes. Left: 2D micropatterned colonies of either mESCs or hPSCs show self-organisation and emergence of the germ layer markers. Right: Gastruloids are 3D cell aggregates of either mESCs or hPSCs that display symmetry breaking, self-organisation, convergent extension, and the formation of intrinsic axes (D Dorsal, V Ventral, A Anterior, P Posterior, R Right, L Left. M Mouse and H Human. Figure created using NIAID NIH BIOART^117^.

Gastrulation has been studied for over a century using animal models^35^. However, the emergence of (mouse and human) pluripotent stem cell (PSC)-based embryo models, henceforth gastruloids, has accelerated the discovery of mechanisms that mediate gastrulation. Mouse or human gastruloids can be created using either mouse embryonic stem cells (mESCs) or human PSCs or human induced (hi) PSCs in both 2D and 3D (Fig. 3). Over the last decade, these gastruloids have emerged as a standard methodology in developmental biology and biomedical engineering labs internationally. These models are versatile, tractable, scalable and highly accessible compared to their in vivo counterpart^36^.

### mESC-based embryo models

Nearly fifteen years ago, the first observation of co-ordinated elongation of cellular bodies was reported by Marikawa et al.^37^. They used mouse embryonic carcinoma cells to explore mesoderm formation and axial elongation. This was a precursor to efforts made by developmental biologists using mESCs. In 2014, Turner et al.^38,39^ and van den Brink et al.^40^ reported mESC aggregates capable of exhibiting self-organisation, convergent extension, and axial patterning. Subsequently, Beccari et al.^41^ reported a robust and reproducible protocol to form “gastruloids”: small cell aggregates (~1 cm at 120 h of culture) capable of self-organisation, symmetry breaking, germ layer formation, and exhibiting axial *Hox* gene segmentation—all without the need for any extraembryonic tissue or maternal nurture. Culture of these 3D gastruloids has dominated the murine context since the seminal protocol was published^41,42^ and has now been widely adopted by labs globally as a preferred alternative to investigating gastrulation in vivo. Another alternative approach was demonstrated by Blin et al.^43^ in the form of 2D mESC micropatterns that displayed gastrulation-like patterning. More recently, Aguilar-Hidalgo et al.^44^ showed how these 2D micropatterns undergo symmetry breaking, opening up new possibilities for 2D mESC gastrulation modelling and experimentation.

### hPSC-based embryo models

Warmflash et al.^45^ and, independently, Tewary et al.^46^ developed a technique to capture gastrulation-like patterns using 2D hPSC micropatterns. Following exposure to Bone Morphogenetic Protein-4 (BMP4) (and NODAL, as used by Tewary^46^ and co-authors), these colonies self-organise with radially segregated ectoderm-like, mesoderm-like, endoderm-like, and extraembryonic cells (radially outwards in that order). A key limitation of these 2D models is that they do not break symmetry. Moris et al.^47^ reported 3D hPSC gastruloids that are capable of breaking symmetry and, thus, demonstrating polarity and more accurately representing human gastrulation. These 3D aggregates display a remarkably similar molecular hardware when compared to the gastrulating human embryo. Using single-cell RNA-sequencing, Tyser et al.^48^ overlaid transcriptomic data of human gastruloids to its in vivo human counterparts. Revealing that human gastruloids robustly form the anterior-posterior axis, much like the human embryo. In addition to close recapitulation of markers of the gastrulating embryo, including the primitive streak, the organiser was also detected. As reviewed recently in Haantjes et al.^21^, gastruloids offer a powerful tool to experimentally dissect distinct developmental processes. However, it is vital that the experimental data obtained must be incorporated into underlying computational models. To obtain a unified understanding of these developmental processes.

### Comparative framework of the hPSC-based embryo models and their utility

The model systems discussed above are transforming our understanding of one of the earliest developmental decision-making events. The ability to explore across species and spatial dimensions the cellular patterning mechanisms which give rise to higher order cell organisation has been particularly revealing, despite the inherent constraints and limits to interpretability. We briefly explore these limitations and discuss biological utility of these models.

### 2D stem cell models

These models are geometrically-confined stem cell monolayers and typically do not recapitulate spontaneous symmetry breaking, though exceptions exist^44^. They clearly lack maternal cues, implantation mechanics, and long-range extraembryonic interactions. Consequently, the morphology of cells differs to their native counterparts, and the functional relevance is not the same^49^: the outer trophoblast-like layer is different than the trophoblast shell present during early post-implantation stage embryo, cells that undergo epithelial-to-mesenchymal transition are not elongated, and there is lack of polarity. Yet, their simplicity, high-throughput design, and accessibility to perturbations (including incorporation of synthetic gene circuits^50^) make them an ineluctable choice for disentangling the multiscale biology of the emergence of the peri-gastrulation markers. Furthermore, the emergence of 2D symmetry-breaking^44^ systems is enabling systematic exploration of the mechanisms mediating symmetry breaking^17,51^. Additionally, 2D systems can be used to explore the mechanical cues^52^ responsible for the emergence of peri-gastrulation patterns and exploring the effects of genetic knockouts. They can also be used for high-throughput screening of drug compounds and toxicology due to their large scalability.

### 3D stem cell models

3D models, on the other hand, canonically display symmetry-breaking and acquisition of polarity. These models undergo dimensional lengthening akin to convergent extension, which highlights synergistic interactions between signalling pathways and cyto-mechanics in these gastruloids. However, these models display “significant variability”^53^ even when consistent techniques are employed^53^. Like their 2D counterparts, they lack implementation mechanics and maternal cues. Their lack of extra-embryonic tissues, however, can be supplemented by inclusion of specified extra-embryonic (XEN) cells. Bérenger-Currias et al.^54^ cultured mouse embryonic stem cells and XEN cells to form XEN-enhanced gastruloids (XEGs), opening the door to investigating how cell-cell interactions in this context inform systems-level patterns. Importantly, genetic perturbations in gastruloids of key pluripotency or developmental genes can help understand the systems-level impact on patterning and subsequent morphogenesis^55,56^. Gastruloids also provide an exciting platform to dissect epigenomic influences on gastrulation-like patterns due to their unique plasticity. Additionally, 3D gastruloids have found utility across a wide scope of biology beyond development. Their increased complexity makes them a valuable alternative for modelling biological processes broadly^54^, including like Rossi et al.^57^, who developed a model of early embryonic heart development by recapitulating a vascular-like network in gastruloids.

### In silico models recapitulating gastrulation-like patterns

Computational or in silico models exploring the mechanisms regulating gastrulation have mostly focused on simulating the impact of chemical signalling and geometrical effects (including size and morphology) on gastrulation-like patterns. Specifically, reaction-diffusion^58^ has been used as the key modelling paradigm to understand the impact of BMP4 activation and autoregulation^59,60^. Essentially, BMP4, upon activation, triggers its inhibitor NOGGIN and autocatalyses itself. Both Etoc et al.^61^ and Tewary et al.^46^ used this paradigm to explain the emerging gastrulation-like spatial patterns. Tewary et al.^46^ further explained how reaction-diffusion can affect these patterns with changing colony shape and size. Etoc et al.^61^ used the model to propose edge sensing as critical to the establishment of the signalling gradients responsible for the gastrulation-like patterns. McMaster et al.^59^ proposed a morphogen concentration-dependent morphogen flux at the colony boundaries to quantify the effective transport dynamics of BMP4 in the colonies. This particular model with “reactive boundaries” has since been used to recapitulate the dynamics of symmetry breaking in small 2D mouse gastruloids^44^. The BMP4-NOGGIN signalling cascade was next extended by Martyn et al.^8^ to include BMP → WNT → NODAL pathway hierarchy in the hPSC context. Both Chhabra et al.^60^ and Tewary et al.^62^ confirmed that these cascades can evolve dynamically. Tewary et al.^62^ further used the model to show how absence of NODAL can yield preneurulation-associated fate.

These initial standalone RD models were followed by the next generation of multi-paradigm models that integrated gene regulatory network models with higher level (cellular and/or microenvironmental) processes. Kaul et al.^17^ created GARMEN (GRN Agent-based Reaction-Diffusion Modelling Environment): a modelling environment that simulated microenvironment gradients as an outcome of interactions between the virtual cells (or agents) that were, in turn, regulated by an internal GRN. Their approach explained how GRNs are responsible for the radially internalising BMP/WNT wave. Discretising GRNs in space, linking GRNs to tissue-level outcomes, and demonstrating the importance of OCT4 in mediating the divergence of mesendoderm represented key outcomes of their work. Overall, they showed 19 pieces of direct validation between in silico and in vitro colonies, which highlights the accuracy and sensitivity of this approach. Grodstein et al.^63^ integrated an RD pattern with a basic GRN to demonstrate how a closed-loop negative-feedback machine can control morphogenic processes. Despite their results being more general and theoretical, they demonstrated how RD patterns with multiple peaks can lay down multiple gradients for positional information. RD patterns have also been observed in 3D human gastruloids^47^. This suggested that gastruloids can perform axial patterning without external spatial cues.

### Barriers to the development of virtual twins of gastruloids

The brief review above captures the arc of the in-silico approaches proposed to investigate the emergence of gastrulation-like patterns, from the reaction-diffusion paradigm to a multi-paradigm architecture that integrated GRNs, agents, and reaction-diffusion models. The latter represents the optimal methodology to developing virtual twins. Given that the emergence of gastrulation-like patterns is dependent on a multitude of factors (regulatory networks, epigenetics, signalling, cellular interactions, system morphology and size, and mechanics), the virtual twin should offer a modular platform to gain multiscale insights into the system. Such a platform can then enable the identification of non-intuitive, emergent trends that are both generalisable (e.g., the role of a gene in mediating a phenomenon) or unique to a particular system (e.g., the systems-level outcome of a gene knock-out or overexpression). Clearly, our review above shows that computational models are progressing in this direction (from reaction-diffusion-based models to GARMEN) and doing so rather rapidly. However, there are some challenges to this.

*First*, biological complexity and, therefore, self-organisation and emergent phenomena depend on the inherent heterogeneity of the system as well as their microenvironment^22^. However, most modelling paradigms ignore this classic hallmark of biosystems: e.g., continuum approaches that rely on bulk phenomena or GRNs that model single cell behaviour. *Second*, biological systems are spatio-temporally dynamic. In fact, an important signature of emergent systems is that they are dynamically relevant^64^: they contain the information that predicts (or leads to) future evolution of the system. While individual mathematical paradigms can handle this aspect to a limited manner (e.g., a PDE-based model with time-dependent boundary conditions), the inbuilt redundancies in biological systems make it challenging to recapitulate this in an integrated model. *Third*, philosophically, a model is an abstraction to explore a question in a reductionist way. This approach also means models are inherently tractable. However, integrated models, if not properly designed, can result in unmanageably numerous sub-components that make mapping outcomes to inputs extremely challenging. Imagine, for example, the impractically large parametric and solution space for a virtual gastruloid with a GRN with 10 nodes (and for simplicity) 50 edges with ternary states (OFF, LOW, HIGH) informing interactions of 5 different virtual cell types (e.g. pluripotent, ectodermal, mesoderm, endodermal, extraembryonic cells), and being contextualised by Turing models^58^ recapitulating BMP4, NODAL, and WNT activity.

*Fourth*, the computational requirement of these models tends to be quite high given the “state explosion” associated with growing GRN nodes and edges, as well as number of components in a discrete model. Although, to a degree, this challenge can be mitigated via use of high-performance computing and/or powerful GPUs, it does limit who can develop/use these models. And, *finally*, these models require robust and comprehensive validation: not just at the systems level (for example, comparing the appearance of Brachyury^+^ cells in silico vs in vitro), but at every decision-making level (e.g. GRN, cellular, microenvironmental). This is the only way to ensure the entire framework is tractable and operating as expected. This requires careful integration of interdisciplinary^65^ computational and experimental teams, and methodical planning of simulations and experiments, which could be compared across multiple levels. We next show how these challenges can be overcome via an agent-driven multi-paradigm approach.

### Developing virtual twins via the agent-driven multi-paradigm approach

#### What is agent-based modelling?

Agent-based modelling (ABM) is a class of discrete mathematical modelling that divides a system into components that can interact with each other based on a rule-set^66^. As Kaul et al.^22^ stated, “*Unlike the continuum approach with a distinct cause-and-effect motif, the agent-based approach relies on interactions—among agents coupled with their environment—to capture and explain macroscopic observables*.” ABM recapitulates the emergence of global patterns based on system heterogeneity and rules-driven agent interactions: an approach that captures cellular biosystems more faithfully vs other paradigms^22^, even demonstrated at the clinical level^67–70^. ABMs are, therefore, ideal to modelling heterogeneous systems made of locally interacting components contextualised by spatial consideration that lead to self-organisation and/or emergent patterns.

But what exactly is an agent? An agent, put simply, is a programme situated in some environment that is capable of autonomous action in that environment to meet its design objective^71^. An agent, therefore, can be a cell, protein or gene, and the design objective can be proliferation, protein modification, or gene regulation. As such, an agent possesses well-defined boundaries and interfaces, can sense and act on its environment (displaying dynamic reciprocity)^72^, and can control its internal state as well as behaviour. Agents can act in anticipation of future goals and respond in a timely fashion to changes that affect their environment^71^. Quantitatively, an agent’s attributes include: a set of inputs (e.g., signalling or spatial cues); a set of outputs (e.g., cell death or protein secretion); a set of states (e.g., pluripotency or epithelial-to-mesenchymal transition); memory variables (e.g., a pluripotent cell differentiating into a Brachyury^**+**^ mesodermal cell); and a set of functions that map input and memory variable to an output and change of memory (e.g., BMP4 and WNT3 exposure to a pluripotent agent differentiating it into a Brachyury^**+**^ mesodermal agent).

ABM is particularly suited to capturing gastrulation-like patterns because it decentralises control, for example, by introducing multiple loci of control^73^. Therefore, decision-making is limited to the agents’ local situation vs some external entity’s perception of the situation. In ABMs, this is achieved via multiple interacting agents, paralleling the gastrulating embryo or gastruloid, where the cell-cell interplay contextualised by the microenvironment^17^ or spatial cues^44^ is responsible for germ-layer emergence. The critical value of this method is that decision-making occurs during run-time, and the user does not have to specify every possible inter-agent link^17,22^, which can be extremely cumbersome, if not practically impossible.

While standalone ABMs demonstrate the potential of capturing emergent outcomes, they cannot sufficiently recapitulate the multiscale biology of development, disease, and or regeneration. For these, we need multi-paradigm approaches, ideally integrated via ABM methods, of which we discuss next.

### What is multi-paradigm modelling?

In his thesis^71^, Kaul introduced the concept of dynamic assimilation: a non-linear, albeit seamless, integration of diverse data structures (continuous, discrete, binary, ternary, ordinal, etc) between the components of a biosystem and its environment to carry out a set of design objectives. Capturing the multiscale biology of development (which entails (epi)genomic regulation, signalling, mechanical alterations, cellular differentiation, growth) requires computational methods capable of dynamically assimilating information^74^. Multi-paradigm models that integrate more than one distinct computational modalities can help achieve this. However, this is not straight-forward to achieve.

In the context of gastrulation, the diversity of the computational modalities that can be used to simulate the various decision-making hierarchies and the resulting data structures makes their coupling a barrier. For example, Boolean GRNs are inherently devoid of spatiotemporal aspects. While ordinary differential equations-based GRNs may partially overcome this issue, their parametric space can be impractically large. On the other hand, continuum models, by definition, assume the system to be continuous, not as a collection of heterogeneous discrete parts, which precludes them from capturing emergent phenomenon. While continuum formulations allow incorporation of spatial and parametric heterogeneity, which enables higher flexibility and accuracy, these models still show limited ability to capture critical emergent patterns in the context of cellular decision-making: lineage-specification, self-organisation, dynamic relevance, etc. Alternatively, discrete models (including ABMs) are impractical when simulating bulk phenomena (where the population-level behaviour takes higher significance), such as reaction-diffusion. In the context of biological emergence, integrating these approaches, therefore, requires an interface modality that can effectively integrate the diverse higher-level (microenvironmental) and lower-level (genomic, epigenomic, etc) data structures.

We have demonstrated (including in the context of gastrulation-like patterns)^17^ that agent-based modelling is optimal for this. This is possible because ABMs allow users to incorporate user-defined data structures. For example, we integrated computational fluid dynamics^75^ with ABM to understand how bioreactor hydrodynamics influence cellular growth^34^. A non-intuitive, emergent finding was that cellular organisation within bioreactors is influenced by local transport phenomenon (itself dependent on bioreactor features), but they, in turn, shape the very same local gradients.

Importantly, ABMs can be coupled with logic models (e.g. Boolean or Ternary GRNs), whilst being integrated with continuous models. We demonstrated this via GARMEN^17^: with GRN, cell activity, and signalling gradients coupled to each other. We created the GRN by mining literature manually and used ternary outputs (OFF, LOW, HIGH), which allowed us to explore a range of perturbations. BMP/WNT pathways were simulated via the reaction-diffusion paradigm. Agents representing the hPSCs acted as the logical interface between the GRN and spatial gradients. We deliberately kept each layer as parsimonious as possible to keep the overall model tractable. With GARMEN, we explored the phase space of peri-gastrulation patterning with signal- or gene-based perturbations. GARMEN revealed that timing of developmental events is quite critical to patterning. For example, it makes a difference if OCT4 is silenced *before* or *during* differentiation. This is significant because it confirmed that the same master GRN can give us significantly different tissues depending on the *nature* of the perturbation and *timing* of the perturbation. This is key to capturing emergence of functions/diseases.

GARMEN yielded two non-intuitive, emergent findings. First, when OCT4 is silenced during differentiation, the virtually colony fails to yield a fully diverged mesendoderm, i.e., (Brachyury) TBXT^**+**^ and SOX17^**+**^ agents were co-localised (vs control), which was confirmed in vitro. Second, simulating gastrulation-like patterning on a donut-shaped colony (with an internal and external edge) revealed that edge sensing^61^ is not required for patterning. In fact, the simulations revealed that despite the thickness of the colony, the peak signal expression is independent of the edge, which was confirmed in vitro. A significant outcome of GARMEN was that it explained how cells decode their position^17^ despite the availability of modest signal gradients. These results highlight the power and potential of multi-paradigm methods.

### Guidelines to develop virtual twins via the multi-paradigm approach

We recommend the following guidelines encompassing the principles of data integration, interoperability, reproducibility, clear assessment metrics, rigorous validation, and trustworthiness. These are aligned with the standards proposed (discussed elsewhere)^76–79^ for digital twins, which require bilateral information flow that links the physical and the virtual counterparts together^80^.

**Fig. 4:**
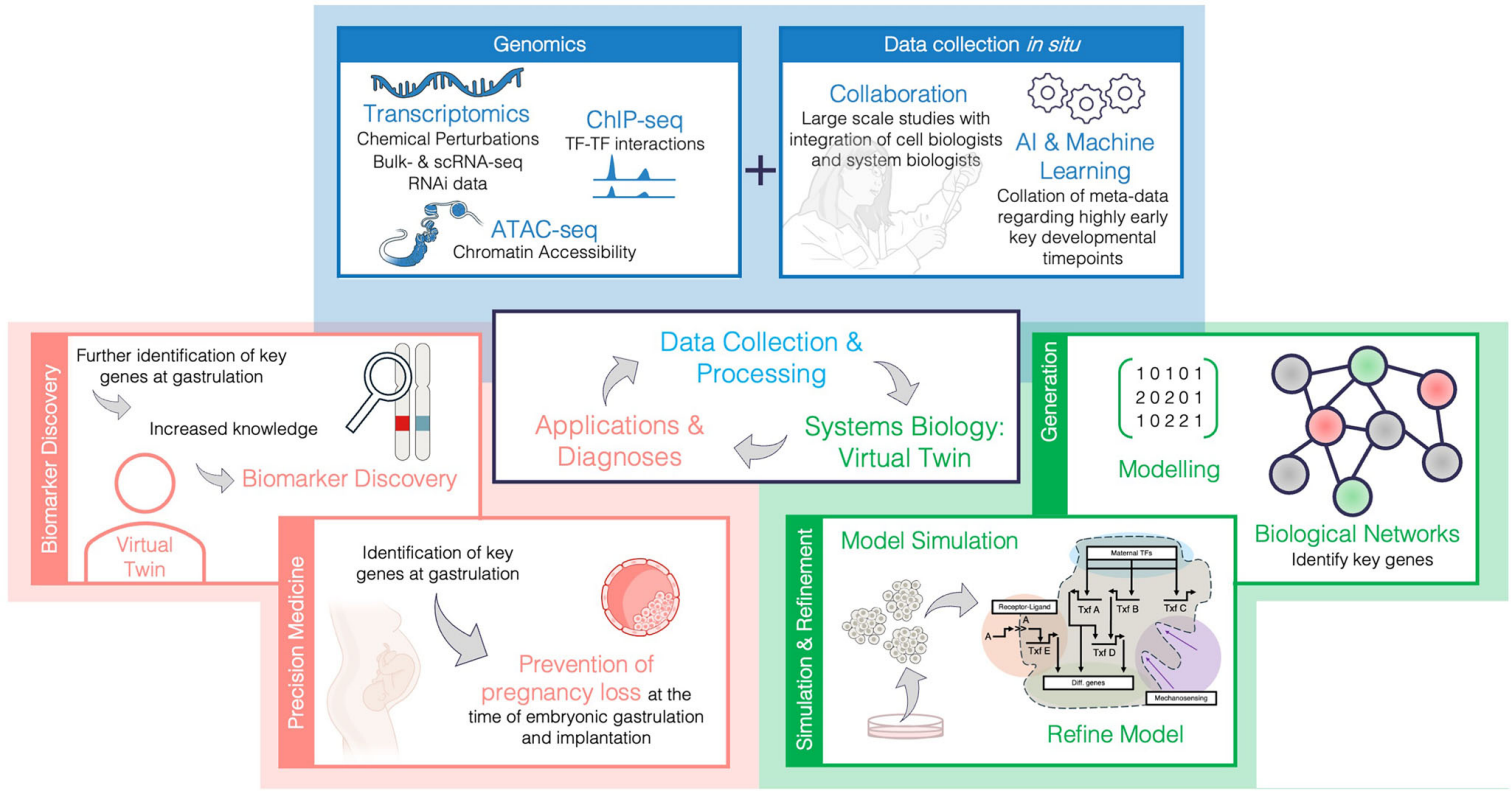
Incorporating biological methods with systems biology to deliver gene regulatory networks for diagnosis, disease, and the formation of a virtual twin. The interdisciplinary collaboration between systems biology, cell biology and genomics holds a powerful key to advance our understanding of early embryonic development with significant societal implications. Figure created using NIAID NIH BIOART^117^.

1. *Keep the model tractable*: Multi-paradigm models afford their users a potentially extensive parametric (and solution) space. This can include, for example, GRN nodes and edges, different forms of agents (e.g. epithelial agents, pluripotent agents, mesenchymal agents, etc), and cues (mechanical and signalling). It is important, therefore, to ensure that the number of components added is sufficient to capture the complexity of the phenomenon being modelled, but with clearly identifiable linkages between the different components. If one cannot map the input to outcomes, the model cannot serve its purpose.
2. *Ensure the various models are in spatiotemporal sync*. This is perhaps most critical to success. While PDE-based models are both space and time sensitive, discrete models lack inherent time, and Boolean models both space and time. Kaul et al.^17^ achieved this by making their ABM time-relevant and aligning its space and time features with the finite elements-based reaction-diffusion model. Essentially, the finite element grid of the RD model provided the spatial coordinates onto which the agents were discretised. Then, the RD model’s outputs were added to the ABM in a time-sensitive manner, such that the 12-h cycle of signal gradient evolution matched the 12 h cycle of agent evolution (growth, differentiation, etc). A master GRN was then introduced in each agent, informing their decision-making based on the specific localised cues the agent was sensing. In this way, the authors discretised >1000 GRNs in space and time, which represented a core novelty of their work.
3. *Validate every hierarchical level of the model independently and, then, collectively*. Kaul et al.^17^ ensured this by validating the GRN, agent layer, and RD model independently. They conducted parametric perturbations by silencing or overexpressing certain genes and inhibiting or triggering signals, followed by comparison of the virtual gastrulation-like patterns with their in vitro counterparts. In all, the authors offered 19 pieces of validation against in vitro observations, offering statistical tests to support in silico predictions. Although this degree of validation was necessitated by the complexity of the model architecture, this approach underscores the rigour of the validation efforts undertaken by the authors.
4. Last, *pair simulations and experiments to enable a direct analogue between perturbations in silico and* in vitro*, so a direct comparison can be made between predictions in silico and outcomes* in vitro (Fig. 4). For example, Kaul et al.^17^ simulated the impact of OCT4-OFF and OCT4-HIGH perturbations in GARMEN. In vitro, these changes were reflected by engineering a human pluripotent stem cell line with transposons that encode genetic circuits for doxycycline-inducible silencing of OCT4 (OCT4-OFF perturbation) and an hiPSC line mimicking the OCT4-HIGH perturbation due to the cell line’s higher OCT4 expression (vs pluripotent stem cell line), confirmed via qRT-PCR.

### Overcoming equifinality and mechanistic degeneracy

In ABMs, multiple rule-sets can yield similar/same emergent patterns: a phenomenon referred to as equifinality^81^. Furthermore, mechanistic degeneracy, i.e. different biological mechanisms yielding same/similar outcomes–for example, the ability of WNT3 and BMP4 to trigger Brachyury expression in hPSC gastruloids^8,17,60^. Both limit interpretability of ABMs and, in turn, ABM-underpinned virtual twins. This challenge can be overcome by adopting a pattern-oriented approach^82^. In the context of multi-paradigm modelling, this entails incorporating details in a “master” rule-set (or GRN) that allows emergence of multiple macroscopic patterns. This is especially an advantage in multi-paradigmatic modelling, given rule-sets need to yield emergent patterns that (statistically) agree with observations at multiple hierarchical levels (see guideline #3 above). For example, GARMEN’s master GRN^17^ predicted that while BMP4 and WNT3 both trigger mesendoderm, the corresponding OCT4 and SOX17 expression profiles for the two cases will vary significantly. This was observed to be the case in vitro (confirmed statistically): OCT4 expression was more localised for BMP4- vs WNT3-triggered gastruloids. Further, SOX17 expression diverged from TBXT in BMP4- vs WNT3-triggered gastruloids.

Fine-tuning can also be achieved via the method of strong inference^83^ where contrasting alternative hypotheses are developed and tested to determine the hypothesis most likely to produce emergent patterns that match biological reality. This includes testing null hypotheses that will not result in emergent properties. Kaul et al.^84^ previously used this approach to investigate how osteoblasts turn into osteocytes, exploring four mechanistic hypotheses^85^ proposed to explain this phenomenon. The four hypotheses were codified into competing rule-sets that each simulated the emergence of a bone nodule. The approach revealed that polarised osteoblasts act synchronously to form osteocytes and that they neither turn off nor slow down matrix secretion. Finally, critical to diagnosing and addressing equifinality is simulating discriminating perturbations, conducting rigorous sensitivity analyses^81^^,^^86^ along with robust experimental analogues to extensively validate predictions.

### Artificial Intelligence (AI), multi-paradigm modelling, and embryology

Any discussion on virtual twins is incomplete without a comment on artificial intelligence (AI) models. AI models are statistical or stochastic, which do not consider the underlying physics or, indeed, biology when predicting outcomes. As such, they can be used in a standalone manner once trained on a sufficient number of datasets. They can, however, also underpin the multi-paradigm models. For example, multiple AI approaches (including deep learning) exist that can help infer GRNs from sequencing data^87,88^. These GRNs can then underpin the activity of virtual cells in the multi-paradigm model. Agentic AI represents the next generation of modelling methods in this revolution, where these “intelligent” agents will be able to identify patterns and unknown unknowns without direct prompts by the user^89^.

Beyond GRN-inference, AI and machine learning models can be employed as meta-models or surrogates to approximate the dynamics of the bulkier multi-paradigm models^90–92^. This is desirable when large numbers of simulations need to be conducted for uncertainty quantification, optimisation, or parameter sweeps, for example^91,93,94^. These surrogates work by inputting parameters and/or initial conditions of the multi-paradigm model and (following training/validation) predicting some or all outputs of the original model^91^. Importantly, machine learning methods, including reinforcement learning, can be applied to refine and even generate agent-based rule-sets in a formal and heuristic manner, as proposed elsewhere^95–101^. Examples also exist of ABMs with embedded neural nets that decide cell phenotype based on inputs from the local environment^102,103^. Agentic AI represents the next generation of modelling methods in this revolution, where these ‘intelligent’ agents will be able to identify patterns and unknown unknowns without direct prompts by the user^89^. For a comprehensive review of how machine learning approaches can support ABMs, the interested reader is directed elsewhere^95^.

However, there is another area where AI can play a critical role. The use of assisted reproductive technologies is becoming increasingly more common. A rise in births from treatments such as IVF now accounts for over 3% of the total births in the UK. This means there is approximately one child conceived via IVF in every classroom^104^. The sophistication of imaging technology (time-lapse imaging) and the advent of computational algorithms have made AI methods^105^ an important part of a clinical embryologists’ toolkit. These algorithms have had remarkable success in predicting “embryo quality” as preselection for transfer in utero. This occurs at multiple stages: moments after fertilisation^106^, monitoring formation of the blastocyst^107^, and even detection of chromosomal abnormalities^108–110^. These tools aid embryologists objectively rank poor or good quality embryos before transfer. It is known that invasive pre-implantation genetic testing (PGT) of the embryo leads to only a live birth in ~14% of embryo transfers^111^. It has been argued that AI algorithms can be used to non-invasively test the underlying embryonic genetic wiring, replacing the invasive PGTs in clinic (provided an accurate, reliable, informed imaging model is available). It is important to note here that the use of AI in clinical settings is not without its controversy. Discussed extensively by Koplin et al.^112^, there is a worrying concern of “dehumanisation”, bias and deskilling due to the use of AI in conducting embryo assessment, including the use of the technology for selecting embryos based on sex or other traits.

## Conclusion

Human gastrulation remains a black box for technical and ethical reasons. Despite its decentralised origins, the process is precisely orchestrated by the flexible interactions between cells and maternal uterine microenvironment, giving rise to the germ layers as well as the body axes (the internal compass), initiating the next developmental iteration, eventually leading to subsequent morpho- and histo-genesis, ultimately resulting in the human form (and function). To capture this dynamic assimilation^71^ of information (signals, mechanical, architectural, cellular, epigenomic) and disentangle how each modality influences cell fate, we proposed that a multi-paradigm modelling approach, such as the one proposed by Kaul et al.^17^, is vital. The preceding decade, especially the gastruloid-based approach, has uncovered a wide array of cell fate-determining genes and pathways that mediate early human development, especially gastrulation. However, it is now time to piece together this information to determine an integrated view of gastrulation and transforms this phenomenon into a white box. The integration of experimental models, systems biology principles, and computer models, in the form of multi-paradigm virtual twins, as discussed above, offers both an ineluctable and transformative opportunity to achieve precisely that.
